# A Wireless Accelerometer-Based Body Posture Stability Detection System and Its Application for Meditation Practitioners

**DOI:** 10.3390/s121217620

**Published:** 2012-12-18

**Authors:** Kang-Ming Chang, Sih-Huei Chen, Hsin-Yi Lee, Congo Tak-Shing Ching, Chun-Lung Huang

**Affiliations:** 1 Department of Photonics and Communication Engineering, Asia University, Taichung 41354, Taiwan; E-Mail: changkm@asia.edu.tw; 2 Graduate Institute of Clinical Medical Science, China Medical University, Taichung 40402, Taiwan; 3 Department of Computer Science and Information Engineering, Asia University, Taichung 41354, Taiwan; E-Mail: abbykayq@gmail.com; 4 Department of Psychology, Asia University, Taichung 41354, Taiwan; E-Mail: zen426@gmail.com; 5 Department of Electrical Engineering, National Chi Nan University, Nantou 54561, Taiwan; E-Mail: tsching@ncnu.edu.tw; 6 Department of Mechanical and Automatic Engineering, Chung Chou University of Science and Technology, Changhua 510, Taiwan

**Keywords:** KW = Posture Stability, wireless tri-Axis accelerator, meditation, leg crossing

## Abstract

The practice of meditation has become an interesting research issue in recent decades. Meditation is known to be beneficial for health improvement and illness reduction and many studies on meditation have been made, from both the physiological and psychological points of view. It is a fundamental requirement of meditation practice to be able to sit without body motion. In this study, a novel body motion monitoring and estimation system has been developed. A wireless tri-axis accelerometer is used to measure body motion. Both a mean and maximum motion index is derived from the square summation of three axes. Two experiments were conducted in this study. The first experiment was to investigate the motion index baseline among three leg-crossing postures. The second experiment was to observe posture dynamics for thirty minute’s meditation. Twenty-six subjects participated in the experiments. In one experiment, thirteen subjects were recruited from an experienced meditation group (meditation experience > 3 years); and the other thirteen subjects were beginners (meditation experience < 1 years). There was a significant posture stability difference between both groups in terms of either mean or maximum parameters (p < 0.05), according to the results of the experiment. Results from another experiment showed that the motion index is different for various postures, such as full-lotus < half-lotus < non-lotus.

## 1. Introduction

Due to the benefits to health and mind there have been many wide-ranging investigations of meditation. Meditation originated in India more than 3,000 years ago and spread worldwide, and now is widely used in medical practice [1]. The first investigative article about meditation was published by Wallace in *Science* in 1970 [2]. He found that there was a significant decrease in heart rate and blood pressure after meditation, and there was increase in skin resistance. These data indicated a relaxation state due to meditation. He also found a significant increase in theta brain wave activity from the frontal brain. There has also been a profusion of meditation research based on brain wave data [3–5]. Newberg found that there was an increase in blood flow volume to the right hippocampus region during meditation sessions [6]. Increasing blood flow in the hippocampus region may be an index of a higher level of concentration due to meditation [7]. The hippocampus region has been found to be related to emotion modulation [8,9]. Other related studies have also demonstrated the stress releasing performance of meditation. Heart rate variability can be an index of autonomous neural activities. The low frequency (LF, 0.04–0.15 Hz) region power is an index of sympathetic nervous activity while the high frequency (HF, 0.15–0.4 Hz) region power is an index of parasympathetic nervous activity. Wu’s data showed that there was a significant decrease in LF power and the ratio of LF to HF, and a significant increase in HF power during meditation [10]. Meditation practice can improve the immune system and reduce illness [11]. Meditation is also found to be a good way to reduce the psychological stress found in a business environment [9].

Meditation is a method of achieving a balance of body and mind. This is achieved at the physical level with fine, soft and long abdominal breathing to keep the brain rested and relaxed. At a psychological level, meditation can be beneficial in achieving stable emotions with sensitive focus and introspection. Chan Masters mention meditation as “staying in meditation with no other consciousness and no other physical characters”. The first important step of meditation is to find one’s inner soul and stay within it during meditation [12], then during meditation inner wisdom will be revealed. The inner wisdom will help oneself to recognize the reality of everything in daily life [12]. Leg crossing is the principle meditation posture. There are three kinds of leg posture when doing meditation. The first type is the full-lotus posture, in which the two legs are crossed over each other; the second type is the half-lotus posture, in which only one leg is placed above the other leg. And the third type is a non-lotus posture, in which two legs are crossed naturally. Five practicing tips are also proposed to achieve a deep meditation state [12]: (1) Sitting like a bell. When doing meditation, the body posture should be very stable, like a big clock standing on a surface; (2) Back as straight as pine trees. The back being straight and not bent is important for breath control to achieve deep meditation; (3) Relaxing the whole body. Remember to relax both the body and mind; (4) Empty consciousness. It is impossible to enter into a meditation state if there are too many wondering thoughts; (5) Focus: To concentrate on specific inner energy chakra without other thoughts. To enter a deep meditation state, the body, mind and consciousness are in a static state. The first practice is to maintain the body without any motion. When body is in a static state, the mind and consciousness will be driven into a static state too. It is not easy to maintain a body position without any slight motion. Strong will power is necessary. This will power can be helpful to overcome physical illness and many unexpected situations in life [12]. An experienced meditation practitioner is required to practice meditation for at least sixty minutes without body motion. Beginners usually feel pain on the foot, leg and back within fifteen minutes when practicing meditation. It is a major lesson to overcome physical pain with will power when doing meditation; therefore sitting without body motion is an important part of training. It is an interesting issue but not one discussed in the previous mediation related literature.

There are some approaches to measuring body motion. The straightforward method is to capture meditation images on several videos. With adequate image processing algorithms, body motion can be determined. The other approach is based on motion capture. With this method, several markers are attached to the body. This method is widely used for animation character simulation [13] and rehabilitation [14]. The third method is by surface pressure sensors. Pressure variation is measured when the meditation practitioner sits on a pressure sensor mat. Body motion and leg shaking during meditation can be shown from the image of the pressure sensor mat. The main disadvantage of the above three methods is cost and measurement system complexity. An economic and rapid motion measurement system is needed. Therefore a tri-axis accelerometer is the candidate of choice. Accelerometers are low cost compared with other measurement systems [15,16]. A tri-axis accelerometer is made from a complementary metal oxide semiconductor (CMOS), and it measures capacitance change that is sensitive to gravity. Gravitational changes are measured in three axes simultaneously and reflected in the output voltages. A tri-axis accelerometer is widely used for physical activity detection [17–19]. In this article, a tri-axis accelerometer is used to measure body motion during meditation. Leg pain is the main cause of motion during meditation. A practitioner will change leg crossing posture to release the pain. The full-lotus leg posture is a more stable body posture than the other two types of leg postures. Experienced meditation practitioners can adopt a full-lotus leg posture through long-term practice. In this article, the degree of body motion between experienced and beginner of meditation practitioners is investigated, with a novel motion index derived from a tri-axis accelerometer.

## 2. Methods

### 2.1. Subjects

All subjects enrolled had practiced meditation. Subjects were required to practice meditation for thirty minutes continuously in a half-lotus posture. Subjects with heart disease and hypertension were excluded. Subjects were required to avoid stimulating drinks such as coffee and alcoholic beverages a day prior to the experiment. The subjects were divided into two groups. The experienced group consisted of thirteen subjects (four male and nine female, with an age range of twenty-two to thirty, mean 24.4 and std 1.9), who had been practicing meditation for more than three years. The beginner’s group consisted of thirteen subjects (eight male and five female, age range nineteen to twenty-four, mean 19.7 and std 1.0), who had been practicing meditation less a year. Both groups were recruited from the same student organization in the same school. Six subjects were chosen to participate in Experiment 1.

### 2.2. Measurements

The experiment was conducted in a light and quiet environment. External noise was excluded to keep subjects in a calm state. A TD1A system (K&Y Lab, Taipei, Taiwan) was chosen for its small size and reasonable cost. It is a wireless system containing one EKG and tri-axis acceleration sensor. Acceleration specification is 0.73 G/cm, and size is 50 × 30 × 10 mm, weight is 11 g. Sampling frequency is 500 Hz. The amplifier was fixed by belt between the abdomen and chest in the same relative position in each experiment measurement. EKG was measured on the left wrist and right ear bone. Recorded data was further analyzed to extract heart rate variability and motion index. The analysis software was coded via MATLAB^®^.

### 2.3. Experimental Procedures

There were two experiments designed in this study. First was the control experiment to determine a baseline of motion index. The second experiment was thirty minutes meditation. TD1A are designed to be tied between chest and abdomen. Experimental details are as follows:

#### A. Experiment 1: Motion-Control Experiment

**Figure 1.**
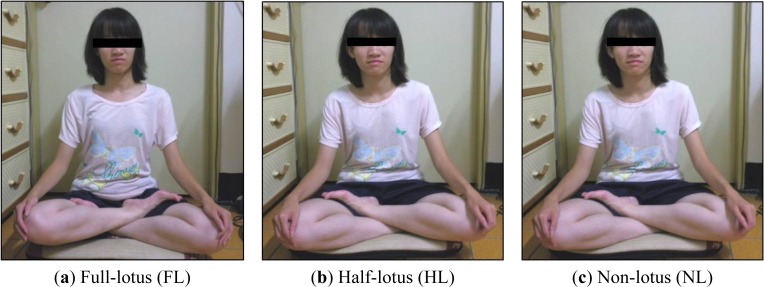
Three leg-crossing postures.

**Figure 2.**
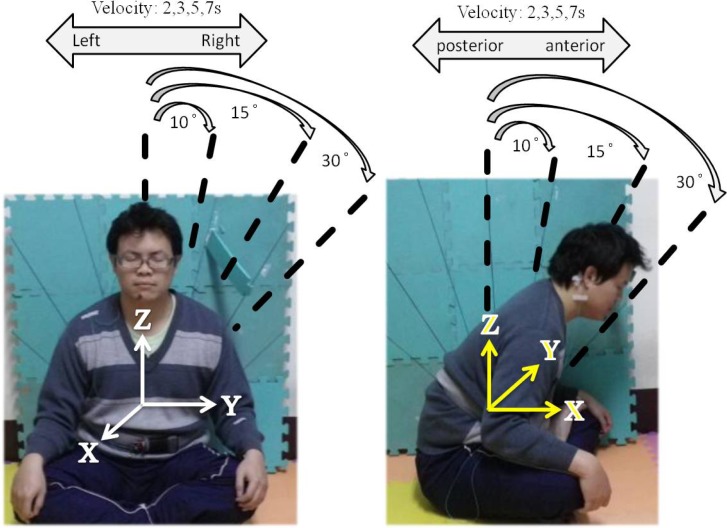
Left-to-right sway and anterior-posterior motion measurement system.

Three possible motion modes were examined; static leg crossing posture (Experiment 1a), leg crossing exchange (Experiment 1b) and controlled body motion (Experiment 1c):

Experiment 1a: Subjects were required to sit for five minutes in each of the three leg postures. One minute’s rest was allowed between two posture experiments. The order of the three postures was a randomly controlled trial for different subjects. Subjects had their eyes closed. TD1A and a video camera were used to record the session. Data are labeled as FL for full-lotus, HL for half-lotus and NL for non-lotus leg postures. The three leg crossing posture models are illustrated in Figure 1.
Experiment 1b: This experiment examined the motion caused by leg posture change. There are four patterns for leg posture change. The first pattern is from full-lotus to half-lotus (labeled as F2H), the second is from half-lotus with left leg above to half-lotus with right leg above (labeled as HL2R). The third pattern is the exchange of left and right to the second pattern (labeled as HR2L). The fourth pattern is from half-lotus to non-lotus posture (labeled as H2N). Subjects had their eyes closed. Bio-signals and video camera were also used to record the session.
Experiment 1c: This experimental examined different body motion factors. Three factors are involved, motion rate, motion angle and direction. Subjects were required to shake seven times within different lengths of time (there are four levels of 2, 3, 5, and 7 seconds), and with different motion angles (there are three angles, 10,15 and 30 degrees) for both directions (anterior-posterior and left-to-right sway) under instruction. Motion angle is marked on a board. Illustration of motion measurement is shown in Figure 2.

#### B. Experiment 2: Thirty Minute Meditation

Subjects were asked to relax until the baseline signal was stable, and then a thirty minute meditation session was begun. Both groups were required to practice meditation with the same half-lotus leg posture and eyes closed. In both experiments the subjects were informed and their consent obtained. IRB was approved by Asia University Medical Research Ethics Committee.

### 2.4. Motion Signal Analysis

Accelerometer data was transmitted via wireless and saved in the host computer as text format and analyzed offline by an analysis program. There are several steps involved in the analysis. The first step is raw data extraction. The second step is preprocessing. By removing the DC component derived from the mode value of each axis, the motion index series is a root-mean-square value of summation of each axis. The final step is feature extraction from the motion index series. Both the mean and maximum value of the motion index series is used:

$${\text{x}}_{i}\left[n\right],i=1,2,3\left(i=1\;\text{for}\;\text{axis}\right)$$

where *n_i_* = length

Step 1:

$${\text{x}}_{i}\left[n\right]={\text{x}}_{i}\left[n\right]|\text{mode}\left(\text{x}\left[n\right]\right)$$

where mode is the median.

Step 2. Root-mean-square:

$${\text{s}}_{1}\left[n\right]=\sum_{i=1}^{3}{\text{x}}_{i}^{2}\left[n\right]$$

$${\text{s}}_{2}\left[n\right]=\sqrt{\frac{1}{2N+1}\sum_{j=-N}^{N}{\text{s}}_{1}\left[n+j\right]}$$

$$I_{1}=\text{mean}\left({\text{s}}_{2}\left[n\right]\right)$$

$$I_{2}=\text{max}\left({\text{s}}_{2}\left[n\right]\right)$$

The corresponding distribution percentage of each axis at a maximum point is also evaluated. Three time sessions, 0–10 min. (phase 1, labeled as P1), 10–20 min. (phase 2, labeled as P2) and 20–30 min. (phase 3, labeled as P3), is divided. Motion index features of three sessions and total experimental sessions are calculated for further analysis. Unit of data is Gravitation (denoted as G).

### 2.5. HRV Analysis

Several frequency domain features of heart rate variability were used. Inclusive of the following parameters:

- HF (%): high frequency power percentage. HF frequency range was between 0.15–0.4Hz on the heart rate variability spectrum.
- LF (%): low frequency power percentage. LF frequency range was between 0.04–0.15 Hz on the heart rate variability spectrum.
- LF/HF: ratio of LF power to HF power.

### 2.6. Statistics

Several statistical methods were used in this study and the IBM SPSS 12.0 software package was used to conduct data analysis. Significance test for the alpha value was set at 0.05.

- Descriptive statistics: Subject’s personal information for experienced and beginner groups, inclusive of heart rate and accelerometer data is represented as mean± standard deviation (mean ± SD).
- T-test: Group differences between experienced and beginner groups with motion index and heart rate variability were compared. The intra-group difference of each meditation phases was also examined via paired t-test. The significance level for the p value was set at 0.05.
- One-way ANOVA: The motion index parameter difference between phase 1, phase 2, and phase 3 in experiment 2 was estimated by one-way ANOVA.

## 3. Results

**Figure 3.**
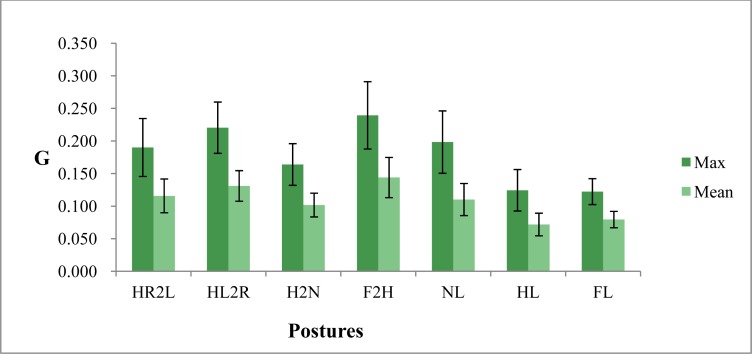
The mean and std motion index for Experiments 1a and 1b.

The motion index is defined as the square summation of the three axes’ acceleration; therefore there are major motion components in either axis. Specific posture change patterns are with corresponding axis acceleration variations. Taking leg crossing posture change as an example, vibration will occur at the moment of changing leg posture. The corresponding effect motion axis is x axis and z axis. The results of Experiments 1a and 1b are shown in Figure 3.

Experiment 1a data showed that non-lotus leg posture had a higher motion value (either mean or maximum motion index) than the other two leg postures. There were no statistical differences between full-lotus and half-lotus leg posture for both motion indices. The mean motion index was below 0.045 G between the three leg postures. The maximum motion index for non-lotus leg posture was averagely around 0.077 to 0.085 G, and the other two leg postures were both below 0.057 G. The non-lotus posture is not more stable than the other postures. The full-lotus and half-lotus postures can stabilize the body and be fundamental in achieving a meditative state. The results from Experiment 1b showed that the mean (0.057 G) and maximum (0.096 G) motion of F2H is larger than that of HL2R, HR2L and H2N. The main motion axes are the x axis and z axis for leg crossing posture change, the motion percentage on each axis is labeled in the corresponding maximum motion index chart. Compared with Experiment 1a, leg crossing exchange caused a larger motion than static sitting postures.

Table 1 organizes the results of Experiment 1C. The main motion axes of left-to-right sway are the x and y axis; while that is y and z axis in anterior-posterior motion. Motion angle is rather more effective than motion rate on motion index value. The higher motion angle is accomplished with a higher motion index value. Generally, anterior-posterior motion caused higher motion than left-to-right sway with the same motion conditions on angle and rate. A reference motion index range for different motion levels can be made from the results of Experiment 1. There are three motion levels for meditation, slight, moderate and severe motion, listed in Table 2. In terms of mean motion index, the range from slight to severe is below 0.057 G, 0.057 to 0.115 G and greater than 0.115 G. In terms of maximum motion index, corresponding ranges are below 0.115 G, 0.115 to 0.215 G and greater than 0.215 G. This result is a useful reference for further examination of the thirty minute meditation period.

**Figure 4.**
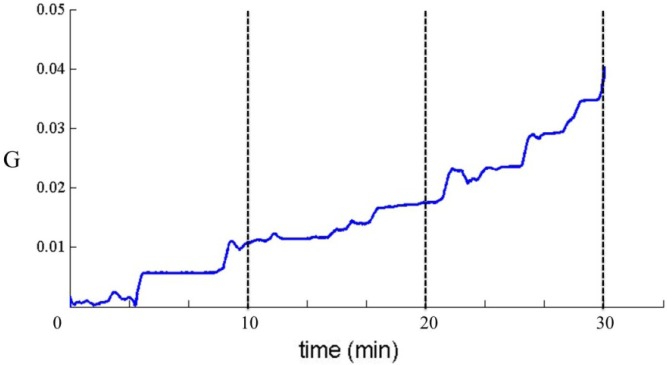
A typical thirty minute motion index series of an experienced meditation practitioner.

Body and leg motion increase as time increase. As shown in Figure 4, a typical motion index time series of a thirty minute meditation is demonstrated. The corresponding Experiment 2 which results for the three meditation phases is organized in Table 3. According to motion level reference shown in Table 2, the motion distributions for slight motion, moderate and severe motion are 80%, 15% and 5% for experienced group, compared to 53%, 32% and 15% for the beginners’ group. As expected, experienced meditation practitioners can be in more static postures than the beginners group throughout the meditation session.

Further detail analyses on different meditation phases find that there are significant differences between both groups on the three phases. Motion index of the experienced groups is lower than that of beginners’ group in the same phase. ANOVA among three phases was applied and the results are shown in Table 4. Both groups’ motion index value was significantly different in the three phases.

The HRV performance of both groups is not as different as in motion. Table 5 lists the HRV value of Experiment 2.

There are only thirty minute and phase 1 LF power value statistically different between two groups. The other intra and inter group analyses did not show any differences between the experienced and beginner groups, and there was also no difference among the three meditation phases.

## 4. Discussion and Conclusions

The Chan Master [12] described meditation as; “when practicing meditation, one must sit and keep the body static without motion. Meditation is a reflection of inner will power. When practitioners can achieve this meditation state with strong inner will power, they can also face their ordinary life with calm, even in tough situations”. That is why keeping the body static without motion is an important part of training and is also an important learning milestone when practicing meditation. The full-lotus leg posture is the most stable way to keep the body static compared to other postures. The main reason is due to the compact contact of legs and buttocks with the ground, as a three point contact. The half-lotus is also acceptable due to its ground contact effect. A non-lotus posture is not suitable for long term meditation practicing. The knee will rise up from the ground with a non-lotus leg posture. Then the body’s center of mass will fall on the buttocks, just like a tumbler. Then a larger body motion would occur with slight body posture adjustment that is mainly due to pain when doing meditation practice. The results of Experiment 1a also confirmed that a non-lotus leg posture has the highest motion index.

The proposed motion classification based on Experiment 1 is very useful. It is a valuable contribution to quantify a baseline of motion index during meditation. With the requirement of economic and simple implementation, a wireless tri-axis accelerometer is a good choice. But there are some limitations to the use of an accelerometer. The accelerometer should be placed with the same orientation in order to keep recorded data with concise directions on three axes. Local body motion due to respiration is also a factor that may interfere with the motion signal. In this experiment the wireless accelerometer sensor is placed on the frontal site between the abdomen and chest. This place can reduce the motion affect due to chest respiration or abdomen respiration by experienced meditation practitioners. In the future, multi-sensors would a good choice to improve the body motion pattern classification, although it will complicate the measurement system. A video camera with the aid of an image processing algorithm is also useful to detect body motion. Now a single sensor is useful to demonstrate the motion performance between experienced and beginners’ group.

Many articles have reported that meditation is beneficial for stress reduction, which can be reflected in parasympathetic activity. HRV parameters measured in this study didn’t show a similar result. Results from Experiment 2 showed that only LF power achieved significance between the two groups, but HF power did not. The possible reason may be that thirty minute meditation practice is still not sufficient for HRV change. The Chan Master also suggested that an hour’s meditation with full concentration, relaxation and without body motion is a necessary condition for deep meditation. Due to a beginner not being able to practice meditation for sixty minutes, this can be a future investigation for an experienced group.

There are three levels of meditation as described by the Chan Master; static state from body, mind and then consciousness. This article developed the body motion monitoring system. This system can be used to monitor body motion variation from beginners, as they increase their meditation practice over several month or years. It can also be used to monitor how long the beginner takes to achieve the half-lotus and even full-lotus leg postures from a non-lotus posture. This system can be used as a “meditation learning performance checking index”. Furthermore, with the aid of other bio-signal measurements, different meditation states may be estimated. Previous studies have investigated meditation from many aspects, but there are few articles about a motion index. Even for the same subject, the lower motion would mean a better meditation state. With the aid of a motion index measurement system, many meditations related studies can be reviewed and novel findings are expected.

## Figures and Tables

**Table 1.** Mean and maximum motion index of Experiment 1C. First label is motion direction, the second is angle and the third is sway number. For example, A_10_2 means left-to-right sway for 7 times with 10 degree in 2 second.

**Data**	**Mean Motion**	**Max Motion**	**X-axis % at Max**	**y-axis % at Max**	**z-axis % at Max**
A_10_2	0.026 ± 0.031	0.041 ± 0.047	0.20 ± 0.27	0.59 ± 0.38	0.21 ± 0.20
A_10_3	0.037 ± 0.004	0.051 ± 0.050	0.22 ± 0.40	0.53 ± 0.37	0.25 ± 0.26
A_10_5	0.031 ± 0.003	0.045 ± 0.037	0.03 ± 0.04	0.43 ± 0.17	0.54 ± 0.19
A_15_2	0.039 ± 0.039	0.056 ± 0.060	0.08 ± 0.15	0.54 ± 0.33	0.39 ± 0.31
A_15_3	0.044 ± 0.050	0.080 ± 0.106	0.15 ± 0.21	0.45 ± 0.35	0.41 ± 0.21
A_15_5	0.044 ± 0.033	0.085 ± 0.081	0.07 ± 0.14	0.59 ± 0.24	0.34 ± 0.18
A_30_3	0.097 ± 0.091	0.156 ± 0.143	0.19 ± 0.30	0.50 ± 0.25	0.31 ± 0.26
A_30_5	0.157 ± 0.187	0.204 ± 0.207	0.13 ± 0.18	0.57 ± 0.33	0.30 ± 0.36
A_30_7	0.104 ± 0.103	0.155 ± 0.156	0.12 ± 0.06	0.59 ± 0.38	0.29 ± 0.40
B_10_2	0.035 ± 0.029	0.054 ± 0.052	0.23 ± 0.20	0.13 ± 0.10	0.64 ± 0.18
B_10_3	0.044 ± 0.042	0.070 ± 0.066	0.13 ± 0.15	0.30 ± 0.29	0.57 ± 0.27
B_10_5	0.042 ± 0.041	0.064 ± 0.060	0.20 ± 0.22	0.04 ± 0.07	0.76 ± 0.26
B_15_2	0.045 ± 0.036	0.072 ± 0.059	0.10 ± 0.14	0.06 ± 0.06	0.84 ± 0.19
B_15_3	0.053 ± 0.041	0.089 ± 0.078	0.06 ± 0.08	0.04 ± 0.04	0.90 ± 0.07
B_15_5	0.053 ± 0.047	0.099 ± 0.081	0.05 ± 0.07	0.11 ± 0.20	0.84 ± 0.20
B_30_3	0.141 ± 0.119	0.273 ± 0.239	0.12 ± 0.05	0.18 ± 0.19	0.70 ± 0.19
B_30_5	0.158 ± 0.124	0.254 ± 0.213	0.07 ± 0.06	0.17 ± 0.19	0.76 ± 0.20
B_30_7	0.185 ± 0.131	0.266 ± 0.216	0.13 ± 0.13	0.16 ± 0.15	0.70 ± 0.25

**Table 2.** References scale of three motion levels (unit = G).

**Parameters**	**Light Motion**	**Medium Motion**	**Severe Motion**
Mean	<0.057	0.057∼0.115	>0.115
max	<0.115	0.115∼0.215G	>0.215

**Table 3.** Statistical and t-test results between starter group and experiment group. Data is represented as average (standard derivation).

**Features^$^**	**Starter Group**	**Experimental Group**	**P Value**
P1 max	0.080 ± 0.080	0.044 ± 0.044	0.011 *
P1 mean	0.050 ± 0.049	0.030 ± 0.030	0.017 *
P2 max	0.114 ± 0.118	0.068 ± 0.072	0.024 *
P2 mean	0.082 ± 0.082	0.051 ± 0.055	0.029 *
P3 max	0.136 ± 0.129	0.070 ± 0.055	0.003 **
P3 mean	0.088 ± 0.083	0.058 ± 0.061	0.029 *
Total max	0.138 ± 0.128	0.075 ± 0.061	0.003 **
Total mean	0.075 ± 0.068	0.048 ± 0.050	0.015 *
L_level %	53 ± 27	80 ± 30	0.025 *
M_level %	32 ± 21	15 ± 23	0.055 *
H_level %	15 ± 19	5 ± 10	0.077

$: P1 max denoted as max motion index for phase 1. L_level % denoted as percentage of time at low motion state for 30 minute experiment. M_level % denoted as percentage of time at medium motion state for 30 minute. H_level % denoted as percentage of time at high motion state for 30 minute.

* þ < 0.05 ;

** þ < 0.01.

**Table 4.** Statistical and ANOVA analysis results for three phases. Data is represented as average (standard derivation).

**Features/Phase**	**P1**	**P2**	**P3**	**P value**
S_mean	0.050 ± 0.049	0.082 ± 0.082	0.088 ± 0.083	0.007 **
S_max	0.080 ± 0.080	0.114 ± 0.118	0.136 ± 0.129	0.005 **
E_mean	0.030 ± 0.030	0.051 ± 0.055	0.058 ± 0.061	0.003 **
E_max	0.044 ± 0.044	0.068 ± 0.072	0.070 ± 0.055	0.005 **

$: S denoted as starter group and E denoted as experimental group.

** þ < 0.01.

**Table 5.** Inter and intra group statistical and ANOVA analysis results for three phases on HRV parameters. Data is represented as average (standard derivation).

	**Starter Group**				**Experience Group**				**Starter *vs.* Experience**		
Level	P1	P2	P3		P1	P2	P3		P1	P2	P3
Statistics	mean ± std			P Value	mean ± std			P Value	P Value		
RR AVERAGE	744.39 ± 82.88	755.94 ± 70.24	745.27 ± 65.44	0.37	766.02 ± 94.95	754.5 ± 99.09	733.51 ± 105.74	0.37	0.54	0.96	0.73
SDNN	189.35 ± 49.44	174.15 ± 58.65	188.71 ± 58.88	0.41	157 ± 91.22	161.69 ± 96.79	162.27 ± 101.44	0.41	0.27	0.69	0.42
RR_DIFF AVERAGE	166.05 ± 76.16	155.25 ± 78.23	174.85 ± 79.95	0.26	139.59 ± 111.49	144.68 ± 109.6	139.65 ± 116.48	0.26	0.48	0.77	0.37
SDSD	247.07 ± 84.64	225.62 ± 94.02	250.26 ± 91.83	0.25	195.61 ± 135.47	202.92 ± 139.11	199.92 ± 142.41	0.25	0.25	0.63	0.29
RMSSD	246.92 ± 84.59	225.48 ± 93.97	250.10 ± 91.77	0.25	195.49 ± 135.39	202.8 ± 139.03	199.81 ± 142.33	0.25	0.25	0.63	0.29
SD1	174.71 ± 59.85	159.54 ± 66.48	176.96 ± 64.93	0.25	138.31 ± 95.79	143.48 ± 98.36	141.36 ± 100.7	0.25	0.25	0.63	0.29
SD2	200.81 ± 47.32	185.51 ± 57.59	198.18 ± 58.31	0.54	169.03 ± 95.9	173.48 ± 103.91	176.63 ± 109.73	0.54	0.29	0.71	0.53
SD1_SD2 ratio	0.86 ± 0.2	0.83 ± 0.23	0.86 ± 0.19	0.25	0.77 ± 0.26	0.75 ± 0.3	0.73 ± 0.29	0.25	0.35	0.5	0.19
VLF	9.03E+08 ± 2.95E+08	1.01E+09 ± 7.22E+08	1.14E+09 ± 1.02E+09	0.68	6.65E+08 ± 4.09E+08	2.55E+09 ± 6.88E+09	3.38E+09 ± 9.19E+09	0.68	0.1	0.43	0.39
LF	2.64E+09 ± 1.1E+09	2.45E+09 ± 1.33E+09	2.87E+09 ± 1.19E+09	0.43	1.44E+09 ± 1.2E+09	1.8E+09 ± 1.83E+09	1.86E+09 ± 1.86E+09	0.43	0.014*	0.31	0.11
HF	5.8E+09 ± 2.98E+09	5.05E+09 ± 3.22E+09	5.57E+09 ± 3.09E+09	0.47	3.94E+09 ± 3.85E+09	4.62E+09 ± 4.25E+09	4.71E+09 ± 4.6E+09	0.47	0.17	0.77	0.58
LF_HF ratio	0.65 ± 0.71	0.57 ± 0.27	0.71 ± 0.59	0.62	0.57 ± 0.43	0.56 ± 0.4	0.8 ± 0.7	0.62	0.71	0.94	0.73
nLF	0.33 ± 0.16	0.34 ± 0.1	0.37 ± 0.12	0.47	0.33 ± 0.13	0.32 ± 0.14	0.37 ± 0.2	0.47	0.93	0.68	0.97
